# Culturing adequate CAR-T cells from less peripheral blood to treat B-cell malignancies

**DOI:** 10.20892/j.issn.2095-3941.2021.0040

**Published:** 2021-08-14

**Authors:** Lu Han, Jian Zhou, Linlin Li, Keshu Zhou, Lingdi Zhao, Xinghu Zhu, Qingsong Yin, Yufu Li, Hongqin You, Jishuai Zhang, Yongping Song, Quanli Gao

**Affiliations:** 1 Department of Immunology, Affiliated Cancer Hospital of Zhengzhou University and Henan Cancer Hospital, Zhengzhou 450008, China; 2 Department of Hematology, Affiliated Cancer Hospital of Zhengzhou University and Henan Cancer Hospital, Zhengzhou 450008, China; 3 Department of Medical Microbiology, School of Basic Medical Sciences, Xinxiang Medical University, Xinxiang 453003, China; 4 The Shenzhen Pregene Biopharma Company, Ltd., Shenzhen 518118, China

**Keywords:** Fewer initial lymphocytes, peripheral blood, CAR-T cells, B-cell malignancy, acute lymphoblastic leukemia

## Abstract

**Objective:**

Chimeric antigen receptor-modified T (CAR-T) cells have shown impressive results against relapsed/refractory B cell malignancies. However, the traditional manufacture of CAR-T cells requires leukapheresis to isolate large amounts of peripheral blood T cells, thus making some patients ineligible for the procedure.

**Methods:**

We developed a simple method for CAR-T cell preparation requiring small volumes of peripheral blood. First, CD3^+^ T cells isolated from 50 mL peripheral blood from patients (B-cell malignancies) were stimulated with immobilized anti-CD3/RetroNectin in 6-well plates and then transduced with CAR-expressing lentiviral vector. After 4 d, the T cells were transferred to culture bags for large-scale CAR-T cell expansion. *In vitro* and animal experiments were performed to evaluate the activity of the manufactured CAR-T cells. Finally, 29 patients with B-cell acute lymphoblastic leukemia (B-ALL) and 9 patients with B-cell lymphoma were treated with the CAR-T cells.

**Results:**

The CAR-T cells were expanded to 1–3 × 10^8^ cells in 8–10 d and successfully killed B cell-derived malignant tumor cells *in vitro* and *in vivo*. For patients with B-ALL, the complete remission rate was 93% 1 month after CAR-T cell infusion; after 12 months, the overall survival (OS) and leukemia-free survival rates were 69% and 31%, respectively. For patients with lymphoma, the objective response rate (including complete and partial remission) was 78% 2 months after CAR-T cell infusion, and after 12 months, the OS and progression-free survival rates were 71% and 43%, respectively. Cytokine-release syndrome (CRS) occurred in 65.51% and 55.56% of patients with B-ALL and B-cell lymphoma, respectively; severe CRS developed in 20.69% of patients with B-ALL and in no patients with lymphoma.

**Conclusions:**

Our novel method can generate sufficient numbers of CAR-T cells for clinical use from 50–100 mL peripheral blood, thus providing an alternative means of CAR-T cell generation for patients ineligible for leukapheresis.

## Introduction

Impressive responses have been achieved in some patients with relapsed/refractory (R/R) B-cell acute lymphoblastic leukemia (B-ALL), chronic lymphocytic leukemia, and B-cell lymphoma after infusion of autologous CD19 chimeric antigen receptor (CAR)-T cells^1–4^. However, despite the clear advantages of this strategy, manufacturing CAR-T cells requires a large amount of starting T cells and complex production procedures, thus precluding the widespread application of CAR-T cells in cancer treatment, particularly in patients in a critical clinical condition.^5,6^

Leukapheresis is the standard method for collecting T cells for CAR-T cell generation, and collecting a sufficient number of T cells is critical^7,8^. However, for patients with certain conditions, such as those with very low peripheral blood platelet counts, heavily pretreated patients, and certain pediatric patients^9,10^, apheresis machines cannot be used to collect peripheral blood lymphocytes (PBLs). For patients ineligible for the traditional CAR-T cell preparation method, new CAR-T cell production methods are needed. Additionally, the actual number of initial lymphocytes required to prepare CAR-T cells for clinical use is unclear. The initial T cell count needed for CAR-T cell preparation may be lower than that obtained through the traditional method and may therefore require lower volumes of peripheral blood than those collected through leukapheresis. Accordingly, the protocol for preparing CAR-T cells requires further optimization to simplify the manufacturing process.

RetroNectin is a chimeric polypeptide of human fibronectin fragments produced in bacteria. It contains 2 functional domains that interact with integrins on target cells. RetroNectin enhances retrovirus-mediated gene transduction by interacting with target cells and virions, thereby placing them in proximity^11^. Additionally, T lymphocytes can be efficiently expanded through stimulation with a combination of immobilized RetroNectin and an anti-CD3 monoclonal antibody (mAb), thereby resulting in higher expansion rates than those with other methods^12–14^. RetroNectin has been used in the CAR-T culture system reported by Kochenderfer et al.^15,16^, and the findings suggest that RetroNectin-activated CAR-T cells can be safely and effectively used to treat B-cell malignancies.

Here, we developed a strategy for CAR-T cell extraction from a small volume of peripheral blood, followed by stimulation with immobilized anti-CD3/RetroNectin. CAR-T cells produced with this method exhibited anticancer effects *in vitro* and in mice. Most importantly, patients treated with these CAR-T cells achieved good clinical results with manageable toxicity.

## Materials and methods

### Cell lines

The CD19^+^ B-ALL cell line NALM6 and the B-cell lymphoma cell line OCI-LY8 (with > 95% CD19 expression) were obtained from the American Type Culture Collection (Manassas, VA, USA). All cell lines were routinely assessed for the presence of mycoplasma contamination through a polymerase chain reaction (PCR)-based method. NALM6 cells were cultured in RPMI-1640/10% fetal bovine serum, and OCI-LY8 cells were cultured in X-VIVO 15 serum-free medium (Lonza, Basel, Switzerland).

### CAR-T cell generation and proliferative activity

The CD19 CAR design includes a single-chain variable fragment (scFv) from the CD19-specific mAb FMC6330 with a (G4S)3 linker between the variable light and variable heavy domains, a CD8 transmembrane region, a 4-1BB costimulatory domain, and an intracellular CD3 domain. Peripheral blood samples (50 mL) were obtained from patients (B-cell malignancies) after written informed consent was obtained. PBLs were isolated with Ficoll-Hypaque (Sigma-Aldrich, St. Louis, MO, USA), and CD3^+^ T cells were isolated through positive magnetic selection (Miltenyi, Bergisch Gladbach, Germany). Cell purity was assessed with a FACSCanto II system (BD Biosciences, San Jose, CA, USA) after staining with anti-CD3-APC (BioLegend, San Diego, CA, USA).

For CD3^+^ T cell stimulation by RetroNectin and the anti-CD3 mAb (both from Takara Bio, Otsu, Japan), a 6-well plate was precoated with 25 mg/mL (5 mg/well) RetroNectin, and 5 mg/mL (1 mg/well) anti-CD3 mAb. CD3^+^ T cells were resuspended at a density of 1 × 10^6^ cells/mL in X-VIVO 15 serum-free medium supplemented with 3% autologous plasma, 1,000 IU/mL interferon (IFN)-γ (Shanghai Kai Mao Biotechnology Co. Ltd., Shanghai, China), and 1,000 IU/mL interleukin (IL)-2 (Shandong Quangang Pharmaceutical Co. Ltd., Shandong, China). The cells were transduced 1 d after activation by centrifugation at 32 °C with lentiviral supernatant supplemented with 1 µg/mL polybrene (Takara Bio). After 4 d, the T cells were transferred to culture bags for large-scale expansion of CAR-T cells. Fresh culture medium containing 1,000 IU/mL IL-2 and 1% autologous plasma was added as appropriate. Cells were expanded and analyzed by flow cytometry until day 10. Cells were counted every 3 d, and expansion was calculated and used to evaluate the number of CAR-T cells.

### Cytokine secretion and cytotoxicity assays

To verify the specific cytotoxic effects of CAR-T cells against CD19^+^ leukemia cells, OCI-LY8 B-cell lymphoma cells were used as targets. Effector and target cells were co-cultured in 48-well plates at a 1:1 ratio (5 × 10^5^ effector cells to 5 × 10^5^ target cells). After 10–20 h of co-culturing, replicate wells were harvested and washed once, stained with 7-AAD and APC-conjugated mouse anti-human CD19 antibodies (BioLegend) for 20 min on ice, and then washed and resuspended in PBS for flow cytometric analysis. The percentage of CD19^+^ cells represented the residual level of target cells. Supernatants were harvested to analyze the production of cytokines, including IL-6, IL-10, IL-2, IFN-γ, and tumor necrosis factor (TNF)-α, with enzyme-linked immunosorbent assay (ELISA) kits (R&D Systems, Minneapolis, MN, USA) according to the manufacturer’s protocols. All samples were analyzed in triplicate and compared against multiple internal standards with standard curves. Data were acquired and analyzed in MasterPlex ReaderFit (Hitachi Solutions America, Ltd., Irvine, CA, USA).

### CAR-T cell phenotype analysis

CAR-T cells were measured on day 10. Cells were harvested, washed in cold PBS, and resuspended at a density of 1 × 10^6^ cells in 100 µL cold PBS containing 5% fetal bovine serum. Cells were incubated with anti-mouse-IgG (Fab specific)-FITC, anti-CD4-PE-Cy7 (clone OKT4), anti-CD8-PerCP-Cy5.5 (clone SK1), anti-CD45RA-APC-Cy7 (clone HI100), anti-CCR7-APC (clone G043H7), anti-PD1-APC (clone EH12.2H7), anti-TIM3-PE (clone F38-2E2), anti-TIGIT-APC (clone A15153G), anti-BTLA-APC (clone MIH26), anti-CD28-PE (clone CD28.2), and anti-4-1BB-PE (clone 4B4-1) (all purchased from BioLegend) for 20 min on ice. Flow cytometry analysis was performed on a FACSCanto II system with CellQuest software (BD Biosciences).

### Transfer of CAR-T cells to NSG mice

Female NOD-SCID IL-2 receptor gamma-null (NSG) mice (6–8 weeks old; Nanjing Galaxy Biopharma Co. Ltd., Nanjing, China) were engrafted with 1.5 × 10^6^ NALM6-luciferase cells *via* the tail vein. After 7 d of tumorigenesis, the mice were intravenously injected with medium (0.9% NaCl + HSA), mock T cells (1 × 10^7^), a low dose of CAR-T cells (CAR-T L: 5 × 10^6^), or a high dose of CAR-T cells (CAR-T H: 1 × 10^7^). CAR-T cells from a small volume of B-cell malignant cancer peripheral blood were stimulated with anti-CD3/RetroNectin. Bioluminescence imaging was performed as previously described^17^. All mice were observed for 2 months. The time of death was recorded for each mouse, and survival curves were generated. For each experimental group, 5 mice were used for data analysis (except for the CAR-T H group, in which 1 mouse died during bioluminescence imaging).

### Clinical trial design

We performed a phase I/II open-label clinical trial (NCT02924753 and NCT03101709) to evaluate the feasibility and safety of CD19 CAR-T cells generated with our method in patients with R/R CD19^+^ B-cell malignancies. The clinical trial was approved by the Ethics Committee of the Affiliated Cancer Hospital of Zhengzhou University, and written informed consent was obtained from the patients. CD19 CAR-T cells were prepared for patient administration with strict quality control. To culture CD19 CAR-T cells from patients with B-cell malignancies (29 B-ALL and 9 B-cell lymphoma), we collected 50–100 mL of heparinized peripheral blood, isolated PBLs with Ficoll-Hypaque, and isolated CD3^+^ T cells through the aforementioned positive magnetic selection method. CD3^+^ T cells (1.2 × 10^7^ for ALL or 2.4 × 10^7^ for NHL; no less than 1.0 × 10^7^) were seeded into 6-well plates precoated with RetroNectin/anti-CD3 mAb and cultured for CD19 CAR-T cell preparation on days −8 to −10 (the first day of CAR-T infusion was set as study day 0; additional information is provided in the **Supplementary materials**). Patients were administered a fludarabine (FLU)-cyclophosphamide-based conditioning treatment for lymphodepletion (25–30 mg/m^2^ FLU on days −5 to −3 and 600–800 mg/m^2^ cyclophosphamide on days −5 and −4). The CD19 CAR-T cells were harvested, and, after 8 to 10 d of culture, were transfused directly into patients in escalating doses over 3 d without any premedication. Patients were observed closely for at least 2 h after administration.

Cytokine release syndrome (CRS) was assessed with a revised grading system^18^. Other toxicity effects during and after therapy were assessed according to the National Institutes of Health Common Terminology Criteria for Adverse Events Version 4.0. Therapy responses were assessed *via* flow cytometry, morphologic analysis, and iconography. CAR DNA copy numbers were assessed through quantitative real-time PCR analysis to evaluate CD19 CAR-T cell expansion and persistence. Genomic DNA was extracted with a QIAamp DNA Blood Mini Kit (Qiagen, Hilden, Germany) from fresh peripheral blood samples after CAR-T cell infusion. Real-time PCR amplification was performed with a pair of primers and Taq-man probes in a Fast 7500 system (Applied Biosystems, USA). Serum concentrations of IL-2, IFN-γ, IL-6, IL-10, and TNF-α were evaluated through ELISA according to the manufacturer’s instructions. All samples were analyzed in triplicate and compared against multiple internal standards with standard curves. Data were acquired and analyzed with MasterPlex ReaderFit (Hitachi Solutions America, Ltd.). We detected CD19^+^ B cells through flow cytometry to monitor patients for the development of B-cell aplasia, which can be used as a pharmacodynamic measure of CD19 CAR-T cell activity.

### Ethical approval

The study was approved by the institutional review board of each participating center (Approval Nos. 2016ct080 and 2016109), and was conducted in accordance with the principles of the Declaration of Helsinki and the International Conference on Harmonization of Good Clinical Practice guidelines. All patients provided written informed consent before study entry.

### Statistical analysis

Statistical analyses were performed in GraphPad Prism 6.0 (GraphPad, La Jolla, CA, USA). Data are presented as the mean ± standard error of the mean. All patients enrolled as of August 2, 2016 were included in the analyses. Overall survival (OS), leukemia-free survival (LFS), and progression-free survival (PFS) were determined through the Kaplan-Meier method. All *P* values are 2-sided, and *P* < 0.05 was considered statistically significant.

## Results

### CD19 CAR-T cells exhibit high gene transfer efficiency, strong proliferative ability, and significant cytotoxic activity

**Figure 1:**
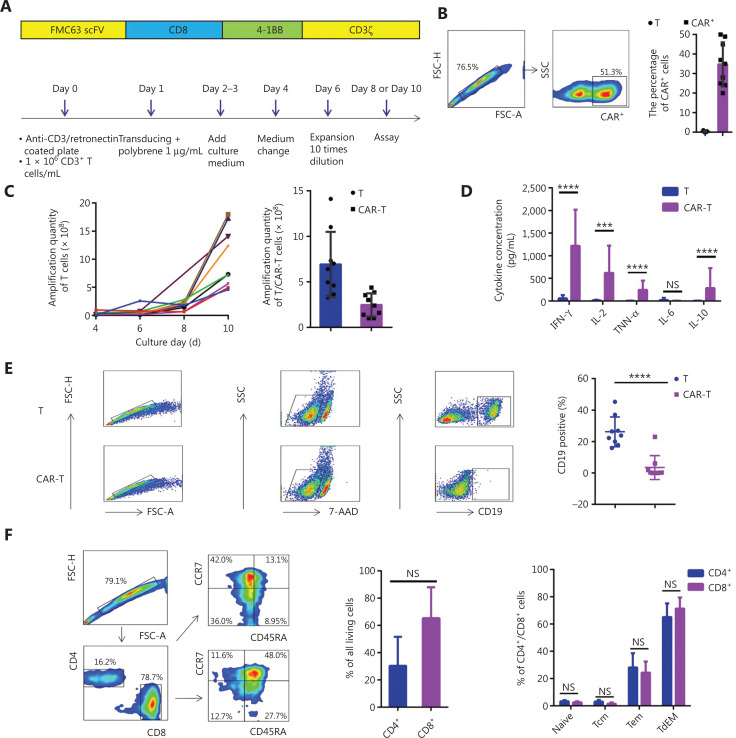
Anti-CD19 CAR design and function. (A) Schematic and culture process for anti-CD19 CAR. The scFv region that recognizes CD19 was derived from the FMC63 mAb. The CAR contained a 4-1BB costimulatory domain and a CD3 ζ T-cell activation domain (top panel). Anti-CD19 CAR-T cells were produced from 50 mL of peripheral blood from patients (B-cell malignancies), and were activated with the anti-CD3 antibody and RetroNectin or anti-CD3/CD28 beads on day 0, then transduced on day 1. The cells were ready for analysis on days 8 through 10 (bottom panel). (B) The gene-transfer efficiencies of cells stimulated with anti-CD3/RetroNectin. Dot plots showing the gene-transfer efficiencies of CAR-T cells from matched samples in 1 representative patient (left panel). Gene-transfer efficiencies of T cells (right panel). (C) Growth curves of T cells (left panel) and the quantity of T cells/CAR-T cells (right panel). (D) Cytokines secreted by T cells or anti-CD19 CAR-T cells, and OCI-LY8 B-cell lymphoma cells were used as targets. NS, no statistical differences; ***P < 0.001, ****P < 0.0001. (E) The percentage of CD19^+^ OCI-LY8 cells, presented as the cytotoxicity of CAR-T cells by flow cytometry after co-culture with CAR-T cells overnight at an E:T ratio of 1:1. Dot plots showing cytotoxicity of T cells or CAR-T cells from matched samples in 1 representative patient (left panel). The cytotoxicity of T cells or CAR-T cells (right panel). ****P < 0.0001. (F) The immunophenotype of CD19 CAR-T cells. Dot plots showing the phenotype of CAR-T cells from matched samples in 1 representative patient (left panel). Immunophenotyping of anti-CD19 CAR-T cells, showing no difference (right panel). Naive: CD45RA^+^ CCR7^+^; Tcm: central memory (CD45RA^−^ CCR7^+^); Tem: effector memory (CD45RA^−^ CCR7^−^); TdEM: differentiated effector memory (CD45RA^+^ CCR7^−^). NS, no statistical differences.

To evaluate the efficacy of our CAR-T cell generation method, we transduced CD3^+^ T cells obtained from 50 mL of peripheral blood and stimulated with anti-CD3/RetroNectin-coated plates with the CAR lentivirus. To promote successful lentiviral vector attachment to T cells, the plate was centrifuged (**Figure 1A**). CAR-T cells were measured on day 10. The gene transfer efficiency with anti-CD3/RetroNectin stimulation was 34.78% ± 3.76%, which was not lower than the values reported in previous studies (**Figure 1B**). Cells stimulated with anti-CD3/RetroNectin expanded approximately 4-fold by day 4 (**Supplementary Figure S1**). The total numbers of T cells and CAR-T cells on day 10 were (6.92 ± 1.19) × 10^8^ and (2.46 ± 0.43) × 10^8^, respectively, indicating considerable numerical expansion (**Figure 1C**). Furthermore, CAR-T cells were successfully produced from all patients on the first attempt, despite the extensive prior treatments that the patients had received.

We next assessed cytokine secretion by CAR-T cells during co-culture with OCI-LY8 cells. Compared with T cells, CAR-T cells secreted higher levels of IL-2, TNF-α, and IFN-γ, and lower levels of IL-6 and IL-10, thus indicating a high degree of proliferation and differentiation after antigen recognition (**Figure 1D**). We assessed the cytotoxicity of the CAR-T cells against OCI-LY8 cells by counting the CD19^+^ cells through flow cytometry, and found that T cells and CAR-T cells exhibited comparable cytotoxicity against OCI-LY8 cells (**Figure 1E**).

We next analyzed the immunophenotypic markers of the expanded T cells after stimulation with anti-CD3/RetroNectin. CD4^+^ and CD8^+^ cells composed 30.25% ± 8.77% and 65.32% ± 9.27% of the population, respectively. CAR-T cells had higher numbers of effector memory (Tem; CD45RA^−^ CCR7^−^) and differentiated effector memory (TdEM; CD45RA^+^ CCR7^−^) cells; however, naïve (CD45RA^+^ CCR7^+^), central memory (Tcm; CD45RA^−^ CCR7^+^), Tem, and TdEM cells did not differ between the CD4^+^ and CD8^+^ populations (**Figure 1F**). Additionally, gating for cells expressing green fluorescent protein (GFP) CAR revealed immunophenotyping results similar to those of ungated CAR-T cells (**Supplementary Figure S2**). CD4^+^ and CD8^+^ CAR-T cells are maintained at certain proportions *in vivo*, which may mediate synergistic antitumor responses^19^. In particular, different subsets of CD8^+^ CAR-T cells, including naïve, Tcm, and Tem cells, exhibit synergistic antitumor effects that persist *in vivo*^19,20^. Moreover, CAR-T cells generated with our method exhibited low expression of the negative checkpoint markers PD1, BTLA, and TIGIT, and high expression of the positive co-stimulatory molecule CD28, thus suggesting that the CAR-T cells that we produced were not depleted T cells^21^. These results indicate the potential of stimulating small numbers of lymphocytes with anti-CD3/RetroNectin for adoptive CAR-T cell therapy.

### Tumor clearance in NSG mice

**Figure 2:**
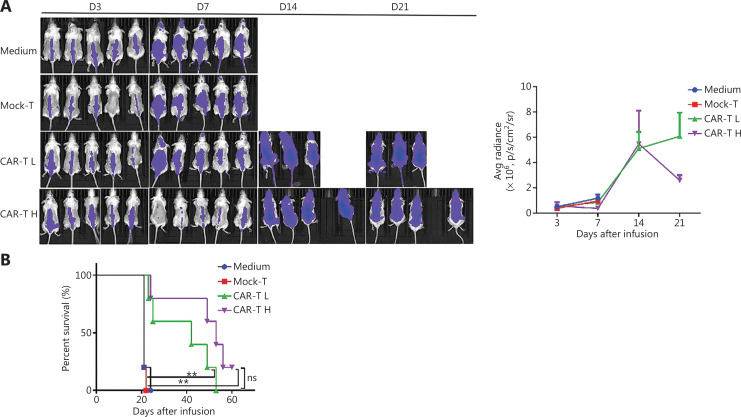
CAR-T cell products from patients exhibit enhanced potency in NSG mice. (A) Bioluminescence analysis of NALM6-bearing NSG mice receiving medium (0.9% NaCl + HSA), 1 × 10^7^ mock-T cells (control), and CAR-T cells 5 × 10^6^ (CAR-T L) or 1 × 10^7^ (CAR-T H). Bioluminescence image analysis (left panel) and changes in mean bioluminescence intensity (right panel) in each experimental group at different time points after treatment. (B) Survival of NALM6-bearing NSG mice treated with CAR-T cells on day 7 after tumor injection. Compared with mock-T cells, CAR-T cells efficiently attenuated leukemia progression and significantly prolonged survival in mice. **P < 0.01.

Because the CD19^+^ CAR-T cells stimulated with anti-CD3/RetroNectin exhibited strong proliferative ability and effective function, we next evaluated their ability to clear tumors *in vivo*. *In vivo* imaging of both the CAR-T L and CAR-T H groups showed that the average bioluminescence signals from the NALM6-Luc tumor cells in both groups were enhanced at 3, 7, and 14 d post-injection. After 21 d, the average bioluminescence signal of NALM6-Luc cells in the CAR-T L group continued to increase, although the increase slowed significantly, whereas the signal in the CAR-T H group decreased significantly. These results indicated that both low and high doses of CAR-T cells exhibited therapeutic effects in tumor-bearing female mice after 21 d of cell therapy, and the high dose was more effective than the low dose (**Figure 2A**). NSG mice treated with mock T cells or medium died within 1 w after injection. Treatment with CAR-T cells efficiently attenuated leukemic progression and significantly prolonged mouse survival (**Figure 2B**).

### Patient characteristics

**Figure 3:**
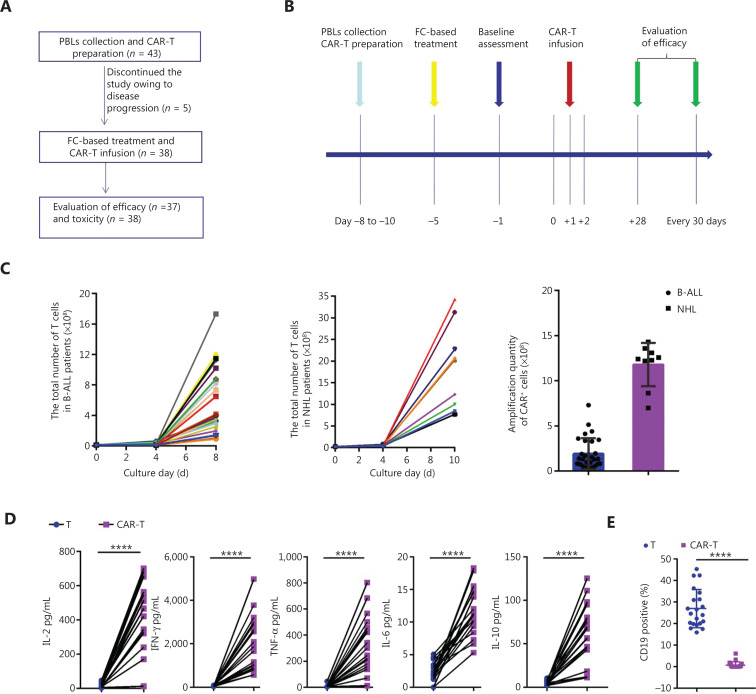
Schematic of the clinical trial of anti-CD19 CAR-T therapy. (A) Patient enrollment flowchart. (B) Clinical treatment protocol. Patients underwent lymphocyte isolation to obtain PBLs on days −11 to −8, with the first day of CAR-T infusion established as study day 0. From days −11 to −8, CAR-T cells were transduced, cultured, and expanded, and patients underwent fludarabine–cyclophosphamide-based lymphodepletion chemotherapy. CAR-T cells were transfused in escalating doses over a period of 3 consecutive days (days 0, +1, and +2) after lymphodepletion chemotherapy. On days −1 and +12, and every 30 days thereafter, bone marrow (BM) examinations were performed. (C) The quantity of CAR-T cells with B-ALL derived from 50 mL of peripheral blood (*n* = 29) and NHL derived from 100 mL of peripheral blood (*n* = 9). (D) Cytokines secreted by anti-CD19 CAR-T cells, and OCI-LY8 B-cell lymphoma cells were used as targets. ****P < 0.0001, *n* = 20. (E) Percentage of surviving CD19^+^ OCI-LY8 cells detected by flow cytometry following co-culture with CAR-T cells or T cells overnight at an E:T ratio of 1:1. CD19 CAR-T cells effectively killed target cancer cells. ****P < 0.0001, *n* = 20.

Between August 2, 2016 and August 31, 2019, 43 patients were enrolled in the study, and peripheral blood samples were obtained. CAR-T cells were successfully generated from all patients. Five patients provided peripheral blood samples but discontinued participation in the study because of disease progression before CAR-T infusion. Therefore, 38 patients with CD19^+^ B-cell malignancies (29 with B-ALL and 9 with B-cell lymphoma; **Tables 1 and 2**) received CAR-T cells (**Figure 3A**). At enrollment, the patients with B-ALL had received a median of 3 previous therapies (range: 2–8) and had a median marrow blast percentage of 49.6% (range: 5.4%–90.8%); moreover, the median leukemia burden in the bone marrow at the time of CAR-T infusion was 32.5% of marrow blasts (range: 1%–92.6%). Of the 9 patients with B-cell lymphoma (8 with diffuse large B-cell lymphoma; 1 with Richter syndrome), 2 had bone marrow invasion, and 1 had received auto-transplantation and radiotherapy. All patients had experienced multiple relapses before receiving CD19 CAR-T cell therapy.

**Table 1:** Patient clinical characteristics of B-ALL (NCT02924753)

ID	Age/gender	Lines of prior therapy	Marrow blasts at enrollment (%)	Marrow blasts before infusion (%)	WBC (10^9^/L)	LYM (10^9^/L)	Peripheral blood collection (mL)	PBMC collection (×10^7^)	CD3^+^ T cell collection (×10^7^)	CD3^+^ T cells for culture (×10^7^)	Transfection efficiency (%)	CAR dose (×10^6^/kg)	CRS grade	Response (day 28)
1	26/M	7	53.2	4	2.56	0.75	50	2.00	1.35	1.2	54	1.5	2	CR
2	24/F	8	46	47.6	17.62	6.49	50	29.25	4.96	1.2	36	2.0	1	CR
3	36/M	5	68	51	1.92	1.29	50	2.59	1.40	1.2	41	4.0	4	CR
4	2/F	3	7	24.2	2.76	0.75	25*	1.70	0.69	0.60	30	1.0	0	CR
5	22/M	6	50.6	48	6.02	2.10	50	3.11	2.29	1.2	50	1.5	1	CR
6	28/F	4	5.4	12.4	4.80	1.48	50	4.38	3.30	1.2	33	1.5	0	CR
7	60/F	3	58.6	74.5	3.51	2.38	50	2.20	1.34	1.2	30	1.0	2	CR
8	17/M	4	71.2	85.5	4.71	1.60	50	5.73	2.00	1.2	55	1.5	5	–
9	54/F	4	7	23.4	5.74	1.47	50	3.38	2.42	1.2	48	1.0	0	CR
10	21/M	4	23	32.5	2.32	0.53	50	2.18	1.36	1.2	52	1.0	0	CR
11	26/F	3	15	21.0	4.15	1.25	50	3.48	2.64	1.2	89	1.0	0	CR
12	6/F	4	60.3	58.2	3.27	0.79	50	3.62	1.33	1.2	20	1.0	0	CR
13	54/F	3	17.8	10	4.84	1.11	50	3.30	2.58	1.2	8	1.5	0	CR
14	15/M	4	6.4	11.6	7.07	1.78	50	2.00	1.84	1.2	31	1.0	1	CR
15	14/M	5	50.8	47.2	3.63	0.63	50	4.85	2.38	1.2	40	1.0	3	CR
16	14/F	2	28.6	4	7.71	1.19	100	3.45	2.11	1.2	19	1.0	1	CR
17	29/M	3	25	9.5	3.3	1.13	50	2.90	2.20	1.2	16	1.0	1	CR
18	24/F	3	77.2	57	65.56	9.46	50	9.79	3.42	1.2	8	1.0	1	CR
19	5/F	3	66	11.52	10.29	2.31	50	7.86	1.99	1.2	40	1.5	1	CR
20	9/F	3	71.25	15.83	2.48	1.04	50	2.50	1.65	1.2	40	1.5	1	CR
21	35/F	5	49.6	3.8	5.55	1.43	50	2.89	1.57	1.2	15	1.0	0	CR
22	11/F	3	48.6	65.2	5.26	1.06	50	2.40	1.78	1.2	35	1.5	3	CR
23	17/F	3	56.6	92.6	4.23	2.47	50	9.25	7.01	1.2	38	1.5	0	CR
24	13/M	4	9.0	81.2	3.44	0.50	50	2.01	1.33	1.2	60	1.5	3	PD
25	15/M	3	22	53	10.49	1.39	50	17.25	4.20	1.2	8	1.5	2	CR
26	31/M	3	52.8	14	1.14	0.63	50	2.70	1.43	1.2	10	1.0	0	CR
27	2/M	2	90.8	60.05	4.99	0.57	40*	3.65	0.80	0.8	60	1.5	1	CR
28	44/M	5	16.6	1	2.31	0.91	50	1.60	1.20	1.2	13	1.5	1	PD
29	32/F	3	70	71.6	6.09	1.72	50	12.38	4.43	1.2	70	1.5	3	CR

All patients were followed up until February 5, 2018; M, male; F, female; C, chemotherapy; R, radiation therapy; T, hemopoietic stem-cell transplant; CRS, cytokine release syndrome; PBMC, peripheral blood mononuclear cell; WBC, white blood cell, normal range (3.5–9.5) × 10^9^/L; LYM, lymphocyte count, normal range (1.1–3.2) × 10^9^/L; CR, complete remission; –: No efficacy assessment due to death; *Reduced blood collection because of the young age of the child and the need for fewer cells.

**Table 2:** Patient clinical characteristics of B-cell lymphoma (NCT03101709)

ID	Age/gender	Lines of prior therapy	Previous treatment	Marrow blasts before therapy	Lymphocyte depletion	WBC (10^9^/L)	LYM (10^9^/L)	Peripheral blood collection (mL)	PBMC collection (×10^7^)	CD3^+^ T cell collection (×10^7^)	CD3^+^ T cells for culture (×10^7^)	Transfection efficiency (%)	CAR dose (×10^6^/kg)	CRS grade	Response (2 months)
1	52/M	6	C	Y	FC	8.43	3.61	100	6.26	3.16	2.4	37	3.0	1	CR
2	39/F	3	C	N	FC	2.54	0.76	100	5.30	2.70	2.4	31	2.0	0	PR
3	35M	3	C	Y	FC	6.15	1.74	100	6.88	4.69	2.4	42	1.5	2	CR
4	60/F	3	C	N	FC	3.25	0.85	100	4.90	2.60	2.4	45	1.5	0	PD
5	51/F	3	C, R, T	N	FC	5.45	0.49	100	3.95	2.13	2.0	30	1.5	0	CR
6	62/M	4	C	N	FC	2.66	0.83	100	4.96	2.66	2.4	20	1.0	0	PD
7	58/F	4	C	N	FC	2.52	0.75	100	7.33	4.90	2.4	60	2.0	2	PR
8	49/M	3	C	N	FC	11.60	0.91	100	8.00	3.46	2.4	45	1.5	1	CR
9	38/M	3	C	N	FC	4.35	1.47	100	7.40	5.30	2.4	39	1.5	1	PR

All patients were followed up until February 5, 2018; M, male; F, female; C, chemotherapy; R, radiation therapy; T, hemopoietic stem-cell transplant; CRS, cytokine release syndrome; PBMC, peripheral blood mononuclear cell; WBC, white blood cell, normal range (3.5–9.5) × 10^9^/L; LYM, lymphocyte count, normal range (1.1–3.2) × 10^9^/L; FC, fludarabine, cyclophosphamide; N, no; Y, yes; CR, complete remission; PR, partial remission; PD, progressive disease.

### Generation, characterization, and ***in vitro*** anti-leukemia activities of anti-CD19 CAR-T cells derived from 50–100 mL of peripheral blood

After CAR-T generation and infusion into the 38 patients (**Figure 3B**), the median transduction efficiency was 41.2% (range: 20%–60%). Total T cells and CAR-T cells achieved considerable numerical expansion after 8–10 d in culture, with CAR-T cells reaching 1.90 ± 0.32 × 10^8^ cells on day 8 (in patients with B-ALL, from 50 mL of peripheral blood) and 7.32 ± 1.36 × 10^8^ cells on day 10 (in patients with B-cell lymphoma, from 100 mL of peripheral blood; **Figure 3C**). CAR-T cells also secreted higher levels of several cytokines, including IL-2, TNF-α, IFN-γ, IL-6, and IL-10, than T cells (**Figure 3D**). *In vitro* cytotoxicity assays revealed robust CAR-T activation and significant antitumor activity against CD19^+^ tumor cells (**Figure 3E**). Routine screens of the CAR-T cells for fungi, bacteria, mycoplasma, chlamydia, and endotoxin before infusion were negative. The patients received CAR-T infusions at doses of 0.50 × 10^6^/kg to 4 × 10^6^/kg, which are similar to the CAR-T doses used in previous studies^22^. The release criteria are provided in the **Supplementary Appendix**.

### Induction of remission after CD19 CAR-T infusion

**Figure 4:**
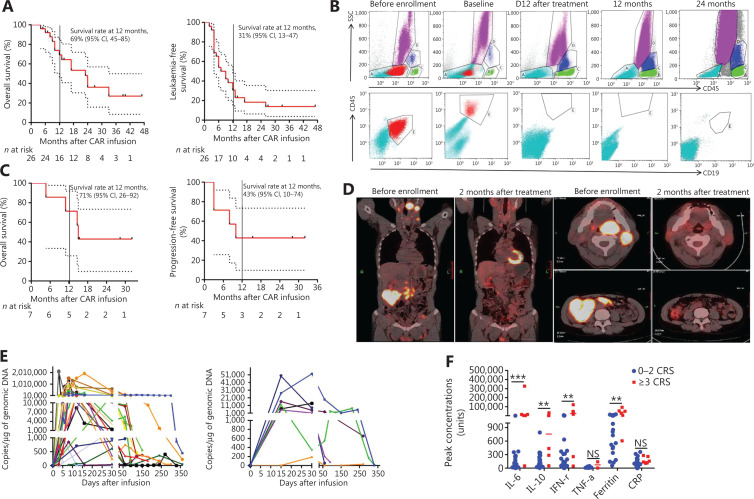
Clinical activity after anti-CD19 CAR-T cell infusion into patients. (A) Prognosis of patients with B-ALL after CD19 CAR-T cell infusion. Kaplan-Meier plot showing 69% (95% CI, 45–85) OS probability at 12 months for 26 patients enrolled (left panel). The LFS rates were 31% (95% CI, 31–47) at 12 months (right panel). All remaining patients were leukemia free, except for patients 9, 12, and 21, who underwent subsequent HSCT. (B) Flow cytometry analysis of MRD by flow cytometry in patient 1 after 24 months of remission. No abnormal B-cell-related tumor cells were found in re-examination. (C) Prognosis of patients with B-cell lymphoma after CD19 CAR-T cell infusion. Kaplan-Meier plot showing OS and PFS rates of 71% and 43%, respectively, at 12 months. (D) PET/CT scans demonstrating CR after CD19 CAR-T treatment for patient 8, who had received chemotherapy for refractory diffuse large B-cell lymphoma with extensive liver and partes oralis involvement. (E) CAR-T cell persistence in the blood from patients with B-ALL (left panel, *n* = 28) and B-NHL (right panel, *n* = 9). Measurements of gene-modified T cells in peripheral blood, as assessed by quantitative real-time PCR. The horizontal line at 5 copies/µg DNA represents the lower limit of quantification of this assay. Data at the first time point were obtained before infusion of CD19 CAR-T cells. (F) Peak concentrations of serum IL-6, IL-10, IFN-γ, TNF-α, ferritin, and CRP after CAR-T cell infusion (median) in B-cell malignancies with CRS grade 0–2 (*n* = 32) and CRS grade ≥ 3 (*n* = 6). Units are as follows: cytokines (IL-6, IL-10, TNF-α, and IFN-γ), pg/mL; ferritin, ng/mL; and CRP, mg/L. NS, no statistical differences; **P < 0.01; ***P < 0.001.

We treated 29 patients with B-ALL and 9 patients with B-cell lymphoma with CAR-T cells. Among the patients with B-ALL, 26 (93%) were in morphologic complete remission (CR) 1 month after infusion, 1 died from CRS, and 2 experienced disease progression. The 26 patients in remission were followed up until relapse. Long-term CAR vector copy detection and OS and LFS calculations included only those patients, whereas data on adverse events was collected on all 29 patients. The OS and LFS rates were 69% (95% confidence interval, CI: 45%–85%) and 31% (95% CI: 13%–47%) 12 months after CAR-T cell infusion, respectively (**Figure 4A**). Patient 1 with B-ALL, showed recurrence after 2 hematopoietic stem cell transplantations and achieved CR after treatment with the anti-CD19 CAR protocol, and durable CR was achieved after 24 months (**Figure 4B**).

Of the 9 patients with B-cell lymphoma, 4 achieved CR, 3 achieved partial remission, and 2 displayed progressive disease after 2 months, for an overall response rate of 78%. The remaining 7 patients were followed up until relapse according to the established procedure. After 12 months, the OS and PFS rates were 71% (95% CI: 26%–92%) and 43% (95% CI: 10%–74%), respectively (**Figure 4C**). Patient 8 was diagnosed with diffuse large B-cell lymphoma and had previously been treated with different chemo-immunotherapy regimens, including R-CHOP, R-MTX/IFO/EPI, R-IVAC plus low-dose lenalidomide, and radiotherapy. At the time of enrollment, patient 8 had a high lymphoma burden in the liver and other areas. After treatment with CAR-T cells, patient 8 entered CR; however, his lymphoma returned after 8 months (**Figure 4D**).

### *In vivo* persistence of CD19 CAR-T cells, CRS, and B-cell aplasia

The levels of CD19 CAR-T cells were easily detectable *via* assessment of CAR DNA copies, which reflected high levels of *in vivo* proliferation (**Figure 4E**). We detected high peak proportions of CD19-modified T cells in the 26 patients with B-ALL who exhibited therapeutic responses (median: 132,469.2 copies/µg genomic DNA; range: 613.7–2,135,476 copies/µg genomic DNA) and in the 7 patients with B-cell lymphoma who exhibited a therapeutic response (median: 13,719.36 copies/µg genomic DNA; range: 188.53–52,275.96 copies/µg genomic DNA).

Levels of laboratory markers of systemic inflammation, including C-reactive protein (CRP) and ferritin, increased along with cytokine levels, and were elevated in most patients. Patients who experienced severe CRS appeared to have higher peak levels of IL-6, IL-10, IFN-γ, TNF-α, CRP, and ferritin than those who did not (**Figure 4F**). The incidence of CRS was 65.51% (19/29) and 55.56% (5/9) in patients with B-ALL and B-cell lymphoma, respectively. Severe CRS developed in 20.69% (6/29) of patients with B-ALL and necessitated intensive care with varying degrees of respiratory support. However, CRS was effectively treated with an anti-IL-6-receptor antibody (tocilizumab), dextromethorphan, and plasma exchange.

We used flow cytometry to detect CD19^+^ B cells to monitor patients for the development of B-cell aplasia, which reflects CD19 CAR-T cell function. B-cell aplasia occurred in all patients who exhibited a response to treatment; these patients subsequently received immunoglobulin replacement to maintain IgG levels > 500 mg/dL.

## Discussion

Here, we developed a new method to generate sufficient numbers of CAR-T cells for clinical use from 50–100 mL of peripheral blood. This method should be valuable for patients, in whom the traditional method of using an apheresis machine for PBL collection cannot be applied. Our results also indicate that the initial number of T cells required for CAR-T cell preparation is lower than expected.

The benefit of leukapheresis with an automated blood cell separator is that a single collection can obtain sufficient T cells for CAR-T cell preparation. The average leukapheresis product contains a median of 98 × 10^8^ (9–341 × 10^8^) nucleated cells with a viability of 99.9% (99.6%–100%) in a median volume of 237 mL (136–310 mL)^23^, including at least 1 × 10^9^ T cells. However, CAR-T cell preparation does not necessarily require this number of cells. Usually, 1–5 × 10^6^/kg CAR-T cells are needed for clinical therapy^2,4,16^. Therefore, an 80 kg patient would require a total of 0.8–4.0 × 10^8^ CAR-T cells. When cultured for 10–20 d, CAR-T cells multiply at least 100 times. If a 10% transfection efficiency during CAR-T cell preparation is assumed, the number of initial T cells needed is approximately 0.8–4 × 10^7^. If a patient’s routine blood lymphocyte count is 0.8 × 10^9^/L, then 50–100 mL of blood is sufficient to meet this requirement. Thus, the calculated number of required T cells is significantly lower than the number of T cells collected through leukapheresis. Indeed, we were able to generate sufficient CAR-T cells for all patients in the study from 50–100 mL of blood, even in 2 patients with lymphocyte counts as low as 0.5 × 10^9^/L. These results demonstrate that we can use lymphocytes extracted from 50–100 mL of blood for CAR-T cell preparation instead of separating PBLs with an automated blood cell separator, which often requires the collection of lymphocytes from several liters of blood. This novel method enables more patients, particularly those who are ineligible for leukapheresis, to benefit from CAR-T cell therapy.

One concern regarding the use of smaller volumes of blood is that a lower number of starting T cells for CAR-T cell production might extend the required culture time and consequently result in CAR-T cell differentiation and decreased persistence of efficacy. However, all CAR-T cells prepared in our study were sufficiently expanded by day 10 of culture, a culture duration no longer than that with the traditional method^1,16^. The 28 patients with B-ALL treated with CAR-T cells had a high CR rate after 1 month, and the longest duration of CR is currently over 3 years. These clinical results indicate that CAR-T cells produced with our method display persistent efficacy.

Previous studies have found that T cell stimulation with RetroNectin and anti-CD3 mAb enhances cell proliferation and results in high levels of retroviral transgene expression^14,24^. Preloading the culture medium with RetroNectin removes inhibitory molecules while leaving purified retrovirus bound to RetroNectin, thereby dramatically enhancing gene transfer efficiency^25^. Additionally, we found that gene transfer was not hampered by the use of a lentiviral vector, even when an unpurified vector supernatant was used^14^. In a previous study, we found that RetroNectin induces T cells to enter the cell cycle, thus accelerating T-cell expansion^13^. Freshly isolated peripheral blood mononuclear cells in G0 are poorly transduced with lentiviral vectors^26–28^, thereby suggesting that T cells in a proliferative state are more effectively transduced. On the basis of these observations, we coated 6-well plates with RetroNectin and anti-CD3 mAb to induce T cell activation. We observed an approximately 4-fold expansion over the first 5 d, with good transfection efficiency by day 10. The higher transfection efficiency of anti-CD3/RetroNectin-activated T cells may have been due to increased numbers of actively proliferating cells when the lentivirus was transfected. Thus, on the basis of our results, anti-CD3/RetroNectin activation improves the T cell growth rate and transfection efficiency, which are crucial in the preparation of sufficient numbers of CAR-T cells from a small number of starting T cells.

The long-term efficacy of CAR-T cell therapy depends on the persistence of CAR-T cells *in vivo*. The stronger the proliferative ability of the infused CAR-T cells, the longer the persistence of therapeutic effectiveness^19,29^. The numbers of naïve T cells produced from the stimulation of T lymphocytes with a combination of immobilized RetroNectin and anti-CD3 mAb was higher than those produced with other methods^14^. In this study, CAR-T cells activated by the anti-CD3/RetroNectin system not only worked in a xenograft leukemic mouse model but also achieved good clinical results, with positive responses in patients with B-ALL and those with B-cell lymphoma. Although our study examined a small number of treated patients, the observed clinical efficacy of the CAR-T cells was nearly equivalent to those described in other studies^1,3,30–33^.

The incidence of CRS was 65.51% (19/29) and 55.56% (5/9) in patients with B-ALL and B-cell lymphoma, respectively. Severe CRS developed in 20.69% (6/29) of patients with B-ALL and was effectively treated with anti-IL-6-receptor antibody (tocilizumab), dextromethorphan, and plasma exchange. In a phase I/IIA study of CD19 CAR-T for patients with chemotherapy resistant or refractory CD19^+^ leukemia and lymphoma, all patients had CRS; severe CRS developed in 27% of the patients and was effectively treated with tocilizumab^1^. Thus, the rate of CRS observed in this study was similar to, if not lower than, that previously reported. The clinical implementation of CAR-T cell therapy is heavily reliant on the safety of the procedure, and, similarly to previous reports, the adverse effects that we observed were manageable^1,34,35^.

## Conclusions

In conclusion, we developed a new method to generate sufficient CAR-T cells from 50–100 mL peripheral blood to treat B-cell malignancies. The generated CAR-T cells were effective in patients and produced manageable adverse effects. This method provides an alternative to the traditional CAR-T cell generation method, particularly for patients ineligible for leukapheresis; however, additional high-quality clinical trials with larger sample sizes will be required for further confirmation.
