# Supplementary material for: Culturing adequate CAR-T cells from less peripheral blood to treat B-cell malignancies
Source: Cancer Biol Med. 2021 Aug 14;18(4):1066–79. doi: 10.20892/j.issn.2095-3941.2021.0040 (PMC8610157; doi:10.20892/j.issn.2095-3941.2021.0040)
Supplement: Supplementary file 1 [file cbm-18-1066-s001.pdf]

Supplementary materials

Methods

Preparation of CAR T cells for patients with R/R CD19$^+$ B-cell malignancies

The CD19 CAR T cells were derived from autologous peripheral blood mononuclear cells, and CD3$^+$ T cells were isolated by positive magnetic selection (Miltenyi, Bergisch Gladbach, Germany) according to the manufacturer's protocol. Cell purity was detected with a FACSCanto II system (BD Biosciences, San Jose, CA, USA) and staining with anti-CD3-APC (BioLegend, San Diego, CA, USA). A total of 1.2×10^7 CD3$^+$ T cells for B-ALL or 2.4×10^7 CD3$^+$ T cells for NHL (no less than 1.0×10^7) were separately seeded into 6-well plates precoated with RetroNectin (25 mg/mL, Takara Bio, Otsu, Japan) and anti-CD3 mAb (5 mg/mL, Takara Bio). The cells were transduced 1 day after activation by centrifugation at 32 °C with lentiviral supernatant supplemented with 1 μg/mL polybrene, with a lentiviral vector containing the anti-CD19 CAR sequence (offered by Shenzhen Pregene Biopharma Company, Ltd.). After 4 d, the T cells were transferred to culture bags for large-scale expansion of CAR-T cells. Fresh culture medium containing 1,000 IU/mL IL-2 supplemented with 1% autologous plasma was added as appropriate. Cells were expanded and analyzed by flow cytometry until days 8–10. All cell products were assessed for potency by IFN-γ release and for CAR expression by anti-Fab antibody staining. Release criteria for clinical T-cell products included the following: cell viability ≥ 70%; CD3$^+$ cells ≥ 80%, CD4/CD8 ratio of 0.1–10, CAR expression on T cells ≥ 10%, as measured by anti-Fab flow cytometry; at least 200 pg/mL of IFN-γ release against CD19$^+$ targets in a standard ELISA; endotoxin ≤ 3.5 EU/mL, mycoplasma negative, and bacterial and fungal cultures negative; residual bovine serum albumin ≤ 1 μg/mL; and replication competent retrovirus testing performed by PCR.

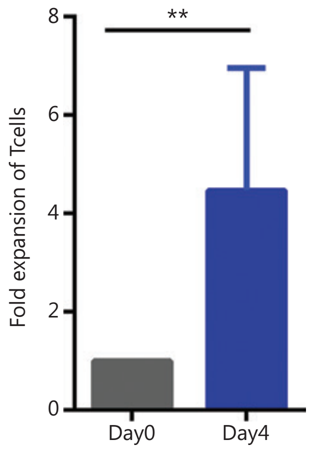

Figure S1 The fold expansion of total T cells stimulated with anti-CD3/RetroNectin on day 4. **P < 0.01.

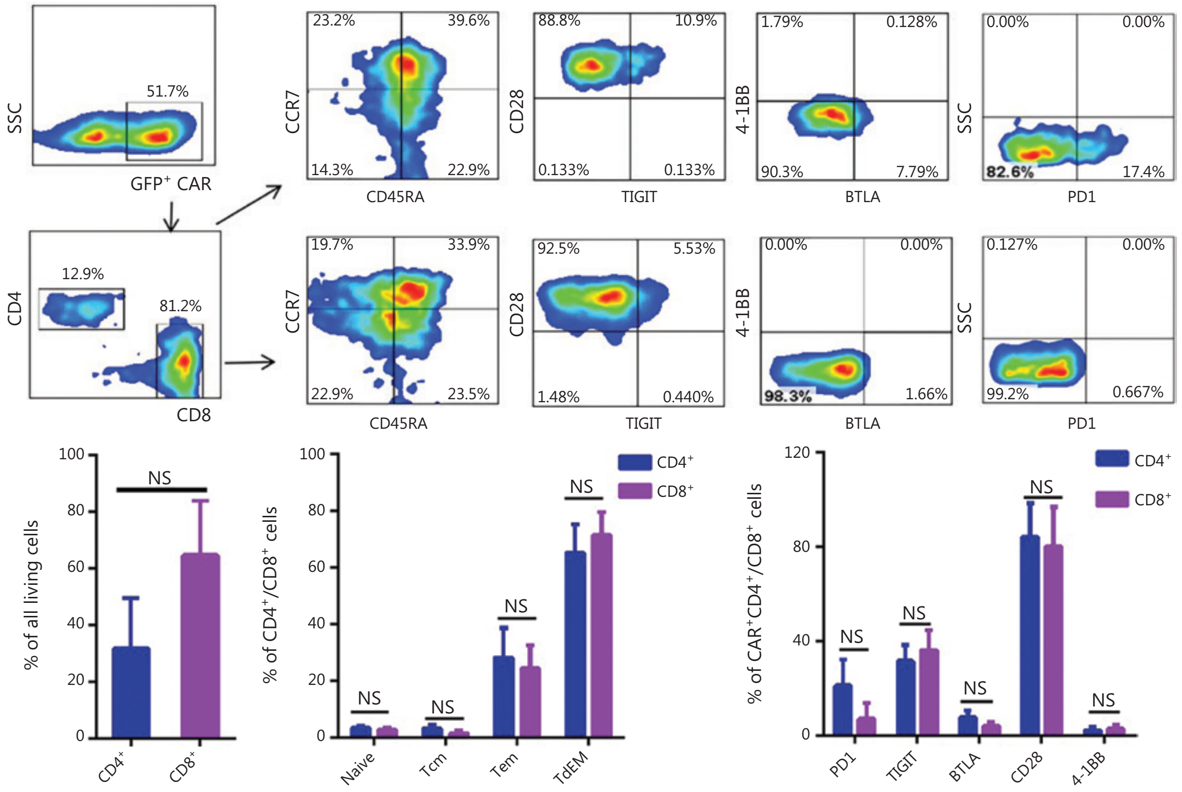

Figure S2 The immunophenotype of CD19 CAR-T cells with green fluorescent protein (GFP). NS, no statistical differences.
